# Primary Sjögren’s Syndrome Presenting With Hypokalemic Rhabdomyolysis, Distal Renal Tubular Acidosis, and Fulminant Necrotizing Vasculitis: A Case Report

**DOI:** 10.7759/cureus.97844

**Published:** 2025-11-26

**Authors:** Ivana Djuran, Bojana Ljubicic, Tijana Azasevac, Nikola Nikolic, Dejan Celic, Violeta Knezevic

**Affiliations:** 1 Department of Nephrology and Clinical Immunology, University Clinical Centre of Vojvodina, Novi Sad, SRB; 2 Faculty of Medicine, University of Novi Sad, Novi Sad, SRB; 3 Department of Emergency Internal Medicine, Emergency Centre, University Clinical Centre of Vojvodina, Novi Sad, SRB; 4 Department of General Surgery, University Clinical Centre of Vojvodina, Novi Sad, SRB

**Keywords:** acalculous cholecystitis, distal renal tubular acidosis, hypokalemia, ischemic colitis, necrotizing vasculitis, rhabdomyolysis, sjögren’s syndrome

## Abstract

Primary Sjögren’s syndrome (pSS) is a systemic autoimmune disease characterized by exocrine gland dysfunction and heterogeneous extraglandular manifestations. Renal involvement, most frequently distal renal tubular acidosis (dRTA), may present with severe metabolic disturbances. Rhabdomyolysis due to hypokalemia and systemic vasculitis are uncommon but potentially life-threatening complications. We report a 36-year-old woman who presented with fever, profuse diarrhea, progressive proximal muscle weakness, and a 10-kg weight loss. Laboratory evaluation revealed profound hypokalemia, hyperchloremic non-anion gap metabolic acidosis consistent with dRTA, and rhabdomyolysis with creatine phosphokinase (CPK) of 42,000 U/L. Electromyoneurography (EMNG) confirmed inflammatory myopathy, while autoimmune serology was positive for antinuclear antibody (ANA), anti-Ro52, anti-SSA, and anti-proliferating cell nuclear antigen (PCNA) with marked hypocomplementemia. Despite supportive therapy, she developed an acute abdomen. Emergency laparotomy revealed perforated ischemic colitis and gangrenous cholecystitis. Histopathology confirmed necrotizing vasculitis of small- and medium-sized arteries. Treatment included continuous renal replacement therapy (RRT) with cytokine hemoadsorption. After surgical stabilization and histopathological confirmation of necrotizing vasculitis, immunosuppressive therapy was initiated according to the European League Against Rheumatism (EULAR) and Kidney Disease: Improving Global Outcomes (KDIGO) recommendations for the management of severe systemic vasculitis with visceral involvement. The patient received intravenous pulse cyclophosphamide (CYC) at a dose of 800 mg (corresponding to a total induction dose of approximately 2.5-3 g (as recommended for remission induction), combined with high-dose methylprednisolone with gradual tapering. Supportive measures included intravenous hydration, Mesna uroprotection, and antiemetic prophylaxis. The patient showed significant clinical improvement, normalization of electrolyte and laboratory parameters, and functional recovery within two weeks. This case underscores two severe extraglandular phenotypes of pSS: dRTA-induced hypokalemic rhabdomyolysis and fulminant systemic necrotizing vasculitis. Both are rarely reported and carry high morbidity and mortality. Recognition of hypokalemia as a potential trigger for rhabdomyolysis and vigilance for vasculitic complications are crucial for timely diagnosis. Multidisciplinary management, including nephrology, immunology, surgery, and aggressive immunosuppression, proved life-saving. pSS may present with atypical and catastrophic extraglandular involvement. Physicians should maintain a high index of suspicion when encountering metabolic disturbances with multisystem features. Early recognition and coordinated intervention can significantly improve outcomes.

## Introduction

Primary Sjögren’s syndrome (pSS) is a systemic, chronic autoimmune rheumatic disease of unknown etiology, primarily characterized by lymphocytic infiltration and immune-mediated destruction of exocrine glands, especially the salivary and lacrimal glands [1]. The resulting sicca symptoms may extend to other mucosal surfaces, including the respiratory tract, gastrointestinal lining, and vaginal mucosa [2]. In addition to glandular involvement, pSS may affect virtually any organ system, manifesting as a spectrum of extraglandular manifestations (EGMs). These are typically classified as non-visceral (e.g., musculoskeletal and cutaneous involvement) or visceral, affecting systems such as the renal, pulmonary, neurological, hematological, gastrointestinal, and cardiovascular systems [3]. Renal involvement is a recognized extraglandular feature of pSS, reported in 16-67% of cases, with distal renal tubular acidosis (dRTA) being one of the more common presentations, observed in approximately 4.3-9% of patients [4]. RTA is characterized by a defect in the renal tubules’ ability to regulate acid-base homeostasis, leading to hyperchloremic, non-anion gap metabolic acidosis, abnormal urine pH, a positive urine anion gap, and disturbances in potassium homeostasis, such as hypokalemia or hyperkalemia [5]. Causes of RTA may include hereditary defects, autoimmune diseases, nephrotoxic medications, and malignancies. Although renal interstitial involvement is observed in up to 30% of pSS cases, it is uncommon for hypokalemia and RTA to be the initial clinical presentation, especially when associated with acute myopathy [6-9]. Hypokalemic rhabdomyolysis, while rare, constitutes a medical emergency that requires urgent diagnosis and targeted treatment. RTA-induced hypokalemia can be asymptomatic or may lead to muscle paralysis, hypocalcemia, and profound metabolic acidosis. Rhabdomyolysis with severe hypokalemia as the first manifestation of pSS has been described only in isolated reports [10]. In the present case, the patient presented with severe hypokalemia and muscle weakness as the first clinical signs, ultimately found to be due to distal RTA secondary to undiagnosed pSS. Notably, she also developed systemic vasculitis, a rare and potentially fatal extraglandular complication. Systemic vasculitis occurs in approximately 10-30% of pSS patients, particularly those who are seropositive for anti-Ro/SSA and anti-La/SSB antibodies, and those with hypocomplementemia [11,12]. The pathogenesis involves deposition of immune complexes, complement activation, and recruitment of neutrophils and mononuclear cells, resulting in vascular endothelial injury and possible occlusion [13-16]. Though intestinal vasculitis is exceptionally rare in the context of pSS, it is clinically significant due to its life-threatening potential [11,15]. Vasculitis in pSS is associated with a poorer prognosis and increased risk of complications, including interstitial lung disease (ILD), lymphoma, and irreversible organ damage. Recent evidence also implicates large-vessel involvement, such as cerebral arteries, which may present as ischemic stroke or transient ischemic attack (TIA) in younger individuals [16]. This case underscores the importance of considering pSS in patients presenting with unexplained hypokalemia, metabolic acidosis, or myopathy, even in the absence of classic sicca symptoms. Furthermore, it highlights the potentially aggressive course of systemic vasculitis in pSS and the critical need for early recognition and aggressive immunosuppressive treatment to mitigate severe complications [2-4,10,17].

## Case presentation

A female patient, with prior medical history including sicca symptoms and axonal sensorimotor polyneuropathy under immunologic evaluation since 2020, presented to the Infectious Diseases Department with a three-day history of high-grade fever, profuse watery diarrhea, generalized fatigue, myalgia, and progressive proximal muscle weakness, which rendered her unable to walk unassisted. The patient also reported a 10 kg unintentional weight loss over the preceding month and noted increasing dysphagia.

On physical examination, the patient appeared chronically ill, emaciated, and dehydrated. Neurological evaluation revealed marked hypotonia, global muscle tenderness, reduced deep tendon reflexes, and diminished grip strength with distal sensorimotor deficits. There were no rashes, joint swelling, or mucosal ulcers except livedo reticularis on the lower extremities. Initial laboratory evaluation is presented in Table 1.

**Table 1:** Laboratory findings CPK: creatine phosphokinase; ALT: alanine aminotransferase; AST: aspartate aminotransferase; GGT: gamma-glutamyl transferase; ALP: alkaline phosphatase; LDH: lactate dehydrogenase; WBC: white blood cell; RBC: red blood cell; C3: component complement 3; C4: complement component  4

Parameter	Result	Unit	Reference range
CPK	>42670	U/L	29-168
ALT	769	U/L	10-40
AST	1258	U/L	10-35
GGT	59	U/L	6-42
ALP	83	U/L	46-116
LDH	1338	U/L	120-246
Total bilirubin	9	µmol/L	3-21
Direct bilirubin	4.5	µmol/L	<5.2
Iron	2.6	µmol/L	9.0-30.4
Urea	1.8	mmol/L	2.5-8.0
Creatinine	30	µmol/L	44-98
Na	133	mmol/L	136-145
Cl	104	mmol/L	98-108
K	2.8	mmol/L	3.5-5.5
Mg	0.95	mmol/L	0.66-1.07
Ca	1.98	mmol/L	2.10-2.55
Uric acid	101	µmol/L	184-464
Total proteins	47	g/L	60-83
Albumin	25	g/L	32-52
WBC	13.66	10⁹/L	4.00-10.00
RBC	3.6	10¹²/L	3.9-5.4
Hemoglobin	102	g/L	120-160
Hematocrit	0.315	L/L	0.370-0.470
Platelets	182	10⁹/L	140-400
Neutrophils %	95.3	%	50-75
Lymphocytes %	1.8	%	20-40
Monocytes %	2.6	%	2-12
C3	0.51	g/L	0.82-1.85
C4	<0.03	g/L	0.15-0.55
RF	103	IU/mL	<30
CRP	146.4	mg/L	<5.0

Urinalysis revealed light turbidity, alkaline urine (pH 8,5) despite the presence of systemic metabolic acidosis, with proteinuria, and absence of glycosuria, with erythrocytes and leukocytes in the sediment (Table 2).

**Table 2:** Urinalysis RBC: red blood cell

Parameter	Result	Reference/comment
Appearance	Slightly cloudy	Normal: clear
Color	Light yellow	Normal: yellow
pH	8.5	Normal: 4.5-8
Specific gravity	<1005	Normal: 1010-1025
Protein	+	Normal: negative
Glucose	-	Normal: negative
Ketones	-	Normal: negative
Hemoglobin	++	Normal: negative
Leukocyte esterase	-	Normal: negative
Nitrites	-	Normal: negative
Ascorbic acid	-	Normal: negative
Sediment-RBC	20-50	Normal: <5/field

The patient had a non-anion gap metabolic acidosis consistent with type I (distal) renal tubular acidosis (dRTA). Immunoserology findings are presented in Table 3.

**Table 3:** Immunoserology findings PCNA: proliferating cell nuclear antigen; ANA: antinuclear antibody; CENP B: Centromere Protein B; ANCA: anti-neutrophil cytoplasmic antibodies

Parameter	Result	Reference range
uRNP/Sm	Negative	Negative
Sm	Negative	Negative
SS-A	+++ (strong positive)	Negative
Ro-52	+++ (strong positive)	Negative
SS-B	Negative	Negative
Scl-70	Negative	Negative
PM-Scl	Negative	Negative
Anti Jo-1	Negative	Negative
CENP B	Negative	Negative
PCNA	++ (positive)	Negative
ds DNA	Negative	Negative
Myositis profile 2	Negative	Negative
ANA (Hep-2)	Intensely positive	Negative
ANA pattern	Speckled, nucleoplasm	/
ANCA	Negative	Negative
Anti-CCP	4.20	Negative

Electromyoneurography (EMNG) confirmed a myopathic pattern with short-duration, low-amplitude motor unit potentials and early recruitment, consistent with active polymyositis. These findings helped exclude neurogenic causes of weakness and reinforced the clinical diagnosis of overlap syndrome with inflammatory muscle involvement.

**Figure 1:**
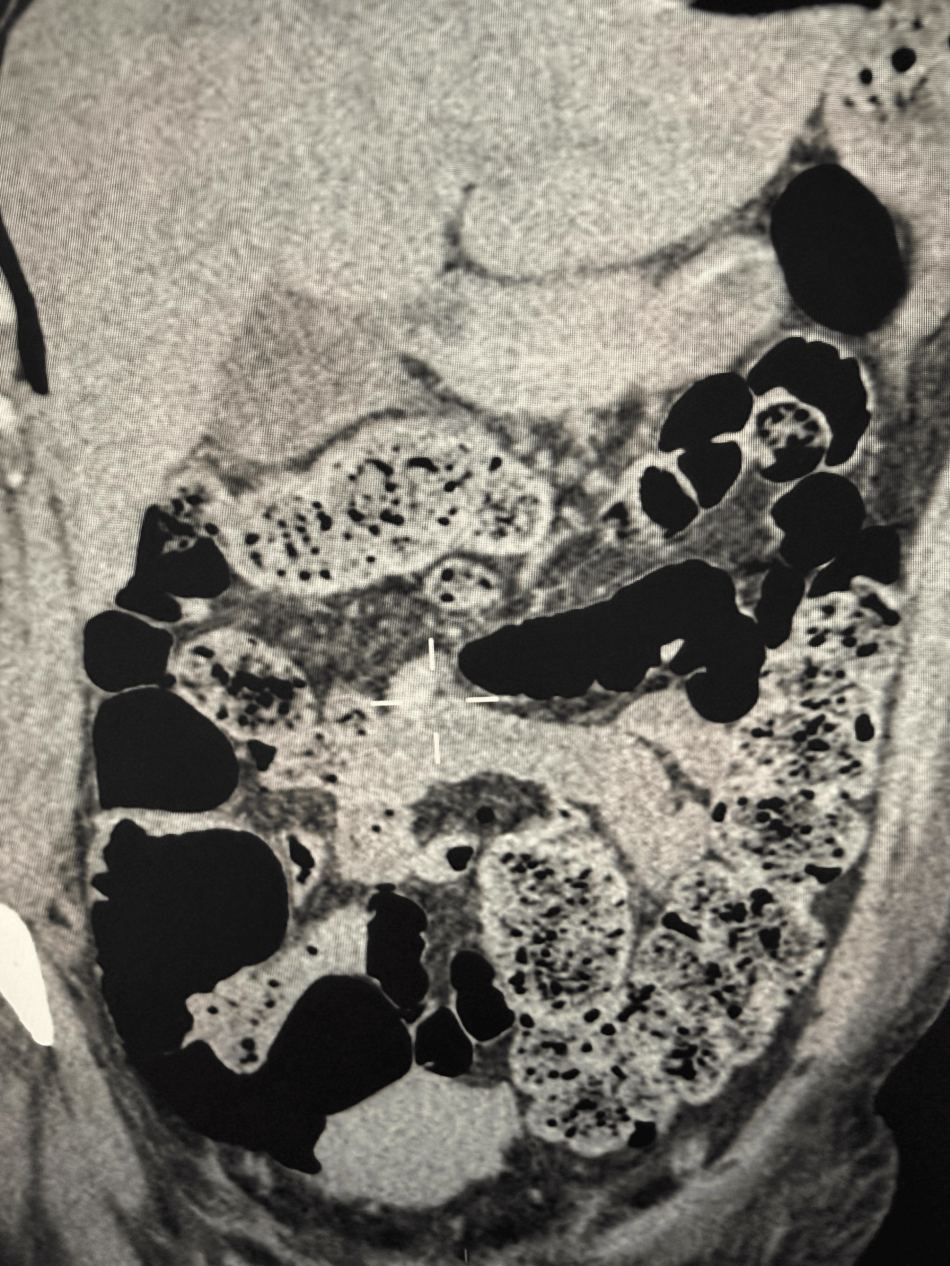
Urgent contrast-enhanced computed tomography (CT) of the abdomen revealing free intraperitoneal air, pericolonic inflammation, and bowel wall thickening

In light of ongoing rhabdomyolysis, worsening metabolic acidosis, hypokalemia, and increased risk for renal injury, continuous renal replacement therapy (CRRT) was initiated. CRRT served a dual purpose, preventing further renal damage and correcting electrolyte and acid-base imbalances. Given the hyperinflammatory autoimmune context, CytoSorb hemoadsorption was concurrently employed to modulate systemic cytokine activity and reduce immune-mediated tissue injury. On day 5, our patient developed another complication, signs of an acute abdomen. Urgent contrast-enhanced computed tomography (CT) of the abdomen revealed free intraperitoneal air, pericolonic inflammation, and bowel wall thickening (Figure 1).

**Figure 2:**
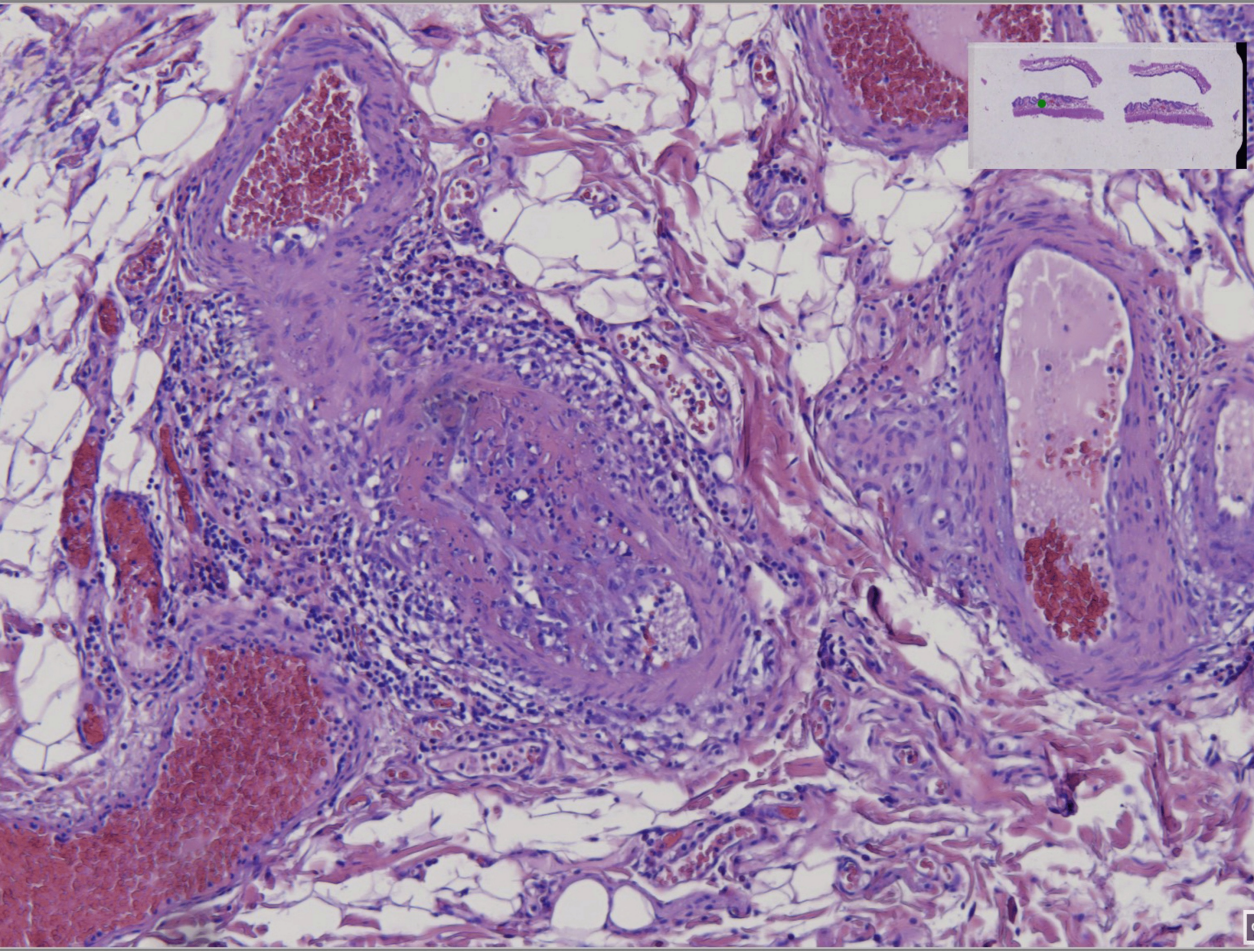
Vasculitis of the colon (HE stain). Histopathological examination of colonic submucosa demonstrates a small- to medium-sized blood vessel with transmural inflammatory infiltrate composed predominantly of lymphocytes and plasma cells, accompanied by fibrinoid necrosis of the vascular wall. Surrounding adipose tissue shows perivascular inflammation and mild edema. Adjacent glandular structures remain intact The image highlights classic morphological changes of vasculitis within colonic tissue [11-13]

Emergency laparotomy uncovered a perforation of the descending colon and inflammatory gallbladder changes. Surgical procedures included left hemicolectomy with terminal ileostomy, cholecystectomy, and peritoneal lavage. Histopathological analysis of the resected colon demonstrated transmural inflammation, crypt architectural distortion, fibrinoid necrosis of medium-sized vessels, and microvascular thrombi, consistent with vasculitis-induced ischemic colitis. Gallbladder histology showed chronic cholecystitis with superimposed gangrenous transformation (Figure 2).

Postoperatively, the patient was transferred to the intensive care unit (ICU) and received broad-spectrum antibiotics (including Tygacil, Tazocin, Imipenem, and Elfonis) and parenteral nutrition. After surgical stabilization and histopathological confirmation of necrotizing vasculitis, immunosuppressive therapy was initiated according to the EULAR and KDIGO recommendations for the management of severe systemic vasculitis with visceral involvement. The patient received intravenous pulse cyclophosphamide (CYC) at a dose of 800 mg (corresponding to a total induction dose of approximately 2.5-3 g, as recommended for remission induction), combined with high-dose methylprednisolone with gradual tapering. Supportive measures included intravenous hydration, Mesna uroprotection, and antiemetic prophylaxis. Enteral nutrition was reintroduced gradually, and the patient underwent early physical rehabilitation due to severe sarcopenia and muscle atrophy.

After postoperative day 14, the patient continued to show gradual and sustained functional recovery. She remained hemodynamically stable with progressive improvement of abdominal symptoms and normalization of inflammatory and renal parameters. Cyclophosphamide therapy was well-tolerated, and maintenance immunosuppression was continued in accordance with the current EULAR and KDIGO recommendations.

The stoma demonstrated adequate perfusion and function without ischemic or infectious complications, with a surgical plan for elective stoma reversal to be considered once the underlying inflammatory process has completely subsided and nutritional status has further improved. The patient was discharged home on hospital day 33, ambulatory with minimal assistance, and enrolled in a structured outpatient rehabilitation program. At the three‑month follow‑up evaluation, she remained clinically stable, without evidence of recurrent vasculitis, gastrointestinal complications, or decline in renal function. She continues with regular rheumatology and nephrology monitoring, with a favorable long‑term prognosis anticipated under continued close follow‑up.

## Discussion

This case illustrates two high-risk extraglandular phenotypes of pSS occurring concurrently: dRTA complicated by hypokalemic rhabdomyolysis and systemic necrotizing vasculitis with catastrophic visceral ischemia. dRTA is the prototypic tubular lesion in pSS and arises from impaired H+ secretion in type A intercalated cells, often accompanied by hypokalemia due to enhanced distal sodium delivery and kaliuresis [3,4]. Severe potassium depletion can cause flaccid paralysis and, rarely, rhabdomyolysis-likely via membrane instability, reduced Na+/K+-ATPase activity, and ischemic susceptibility of muscle [7-10]. Recent reports emphasize that dRTA in pSS may be underdiagnosed, and atypical mechanisms (e.g., AE1 dysfunction) have been described, reinforcing the need for targeted immunosuppression alongside electrolyte and alkali therapy [3,6,9].

Vasculitis in pSS spans a spectrum from cutaneous leukocytoclastic vasculitis to systemic small- and medium-vessel disease, frequently in seropositive patients (anti-SSA) with hypocomplementemia, both present in our patient [11-13]. Cutaneous vasculitis is the most common phenotype and correlates with higher mortality and lymphoma risk; however, visceral involvement remains uncommon but clinically devastating [13,14]. Gastrointestinal ischemia, including mesenteric vasculitis and ischemic colitis, is rare in pSS but carries substantial mortality [15,18]. Our patient’s synchronous mesenteric ischemia and acalculous gangrenous cholecystitis, with histologic evidence of necrotizing vasculitis in both colon and gallbladder vasculature, underscores the potential for widespread vascular injury in fulminant flares [18].

Infectious etiologies were ruled out through extensive microbiological testing, including stool cultures, *Clostridioides difficile* toxin PCR, and multiplex viral panels. The constellation of rhabdomyolysis, hypokalemia, and metabolic acidosis in the context of positive autoimmune serologies supported the diagnosis of overlap syndrome, Sjögren’s syndrome with polymyositis, complicated by extraglandular involvement, including dRTA and systemic inflammation. A striking feature of this case was the marked elevation of creatine phosphokinase (CPK) to 42,000 U/L, suggestive of acute muscle fiber breakdown and rhabdomyolysis in the setting of inflammatory myopathy. Such elevation carries a significant risk for myoglobin-induced acute tubular necrosis (ATN) and warrants close renal monitoring. The presence of livedo reticularis on the lower extremities raised clinical suspicion for underlying vasculitic involvement, which aligned with the emerging picture of a systemic autoimmune flare.

Pathobiologically, immune-complex deposition with complement activation drives neutrophilic infiltration and fibrinoid necrosis of vessel walls, promoting thrombosis and downstream ischemia [1-3]. The association with hypocomplementemia (low C3/C4) reflects complement consumption and signals heightened systemic activity; clinically, it may herald vasculitis and adverse outcomes [11,13,14]. Large-vessel involvement has also been reported in pSS, including cervical/intracranial arteritis presenting with ischemic stroke or TIA in young individuals, emphasizing the need for vascular vigilance beyond the microcirculation [16]. Management hinges on rapid risk stratification and combined modality therapy. In life-threatening visceral vasculitis, current expert guidance and case-based evidence support immediate high-dose methylprednisolone plus a cytotoxic agent (e.g., cyclophosphamide) or B-cell-directed therapy in selected contexts, alongside definitive surgical management for complications such as perforation or gangrenous cholecystitis [6,11,13,14]. In our case, early CRRT mitigated myoglobin nephrotoxicity and corrected acid-base/electrolyte disturbances. The cornerstone of the therapeutic approach in this case aligns with current international guidelines (EULAR 2020, KDIGO 2024), which recommend high-dose glucocorticoids combined with cyclophosphamide or rituximab for remission induction in patients with life-threatening vasculitis manifestations [6,19,20]. Cyclophosphamide was selected due to the need for rapid and potent immunosuppression and drug availability. The use of intravenous pulse dosing allows for a lower cumulative cyclophosphamide burden and reduced risk of leukopenia and urotoxicity, although studies (e.g., CYCLOPS) indicate a slightly higher relapse rate compared to oral therapy [19]. Standard protective measures include Mesna administration and adequate hydration to prevent hemorrhagic cystitis. After induction of remission, transition to maintenance agents, such as azathioprine, methotrexate, or rituximab, is recommended [19]. This case underscores the importance of a multidisciplinary approach (nephrology, rheumatology, surgery, and intensive care) in the management of severe vasculitic complications. Timely initiation of combined immunosuppression and surgical intervention was crucial in ensuring survival and recovery [11,12,16].

## Conclusions

This case serves as a critical reminder that pSS can manifest as a medical emergency through its extraglandular complications, demanding rapid diagnosis, a multidisciplinary approach, and aggressive immunosuppression to prevent a fatal outcome.
